# Social Retribution of Students of Master in Gerontology During the COVID-19 Pandemic: Enhancing Competencies

**DOI:** 10.1093/geroni/igab046.297

**Published:** 2021-12-17

**Authors:** Elva Dolores Arias-Merino, Ma Guadalupe Díaz-González, Neyda Ma Mendoza-Ruvalcaba, Martha Elena Vazquez-Arias, José Rosario Gonzalez-Ulloa

**Affiliations:** 1 Universidad de Guadalajara, Zapopan, Jalisco, Jalisco, Mexico; 2 Desarrollo Integral de la Familia, DIF Zapopan, Zapopan, Jalisco, Mexico; 3 Universidad de Guadalajara CUTONALA, Guadalajara, Jalisco, Mexico; 4 Universidad de Guadalajara, El Palomar, Jalisco, Mexico; 5 Universidad de Guadalajara, Tlaquepaque, Jalisco, Mexico

## Abstract

Introduction: In response to the COVID-19 pandemic older adults were called to lockdown and social isolation. Master in Gerontology (MAGE) in a social retribution action delivered a companion program called “I′m with you, You′re with me”. MAGE competencies encompass those proposed by AGHE. The aim is to analyze competencies acquired by students in gerontology in the companion program during the COVID-19 Pandemic. Method: Participated 16 students of MAGE and 16 older adults selected by their high vulnerability conditions reported by social workers from the Metropolitan Center of Older Adults from DIF-Zapopan city. The program was designed to provide emotional support, was delivered by telephone for 3 months (Ago-Nov 2020). Experiences were obtained through an online-questionnaire, data were analyzed qualitative and quantitatively. Results: Students reported higher development in the competencies: 1) Interactional, that capture the process of knowing and doing across the fields of gerontology, related to stereotypes and discrimination, autonomy and self-determination, and the use of the technology to enhance the communication; 2) Fundamental, that represent the essential orientation to the field of gerontology, related to identify the impact of public policy and the application of intervention strategies and the use of technologies with older adults, families and caregivers; 3) Contextual, related to promote solid social networks for the wellbeing. The meaning of participating in this program was mainly centered in a gratifying professional experience, “small actions that make big gestures”. Conclusion: Gerontologist promote social solidarity through the transference and applying of the knowledge to enhance social development.

